# Disparities in Sleep Health among Adolescents: The Role of Sex, Age, and Migration

**DOI:** 10.1155/2020/5316364

**Published:** 2020-01-28

**Authors:** Maria Jose Miguez, Diego Bueno, Caroline Perez

**Affiliations:** Health Behavior and Policy Initiative, School of Integrated Science and Humanity, Florida International University, Miami, FL, USA

## Abstract

*Background.* Disparities in sleep disturbances have been described in adults; nevertheless, among adolescents, data have yielded conflicting results. Therefore, analyses of our cohort study of 500 urban, normally developed Hispanic adolescents (10–18 years), aim to determine if rates of sleep debt differ between: (a) male and female adolescents, (b) US-born Hispanics and first-generation immigrant ethnic counterparts, and (c) specific activities that these teens trade for sleep. Participants’ weekday and weekend sleep patterns, along with the reasons for sleeping less than the recommended hours were recorded. Standardized surveys were used to gather information regarding sociodemographics, migration, acculturation, and medical history. Using the criteria set forth by the National Sleep Foundation, analyses indicated that sleep deprivation is a pervasive problem, with 75% in the preadolescents and 45% of the late adolescents exhibiting sleep problems. Females slept on average at least one hour less per day than their male counterparts (7 vs. 8 hours). The sleep problems were rooted in several overlapping causes, including use of technology, video games, studying, and employment. Nevertheless, reasons for sleep loss differed by gender and by immigrant status. Multivariable adjusted logistic regression analyses showed that females, US-born teens, and preadolescents had higher odds of being sleep deprived. Pediatricians and sleep experts should be aware of gender-specific causes and responses of sleep problems. Cultural ecological frameworks need to be considered, and clearly indicate that findings may not generalize to youth from other cultural backgrounds.

## 1. Introduction

According to the Center for Disease Control (CDC), up to 70% of U.S. adolescents regularly sleep less than eight hours [1]. This equates to approximately 42 million adolescents suffering sleep deficiencies. The potential impact on society is considerable, given the host of deleterious sequelae due to sleep loss that are common in this age group, including poor school performance, and in extreme cases, death [2, 3].

Gender-specific medicine has gained relevance, as the scientific community continues to recognize gender differences in normal human functioning, pathophysiology, treatment response, and disease manifestation [4]. Nevertheless, most studies focusing on sleep disorders have avoided enrolling females, overlooked sex and gender effects, or applied suboptimal methodology [4]. In addition, sleep research has previously applied findings from adults to adolescents, disregarding the discrete developmental processes of adolescence, and ignoring etiological and behavioral differences [5]. For instance, for most adults, sleep length highly depends on demand of work, accomplishing the tasks needed to keep a household functioning, and fulfilling parental obligations, which are not largely applicable to adolescents. Therefore, it is important to identify which activities they are trading for sleep, so they can be targeted by future interventions.

Analogous pediatric studies have shown mixed results, with some researchers indicating that sex/gender has no influence on sleep, while others argue for significant disparities in prevalence and outcomes [5–8]. Discordance could be due to over reporting in national surveys, (representative of the national population, but lacking objective measures), or convenience sampling (recruiting patients in sleep centers). These conflicting findings and design shortcomings underscore the need for additional research. Consequently, it is important for pediatric medicine to verify the existence of gender differences, and facilitate the development of treatment modalities or adjustments tailored to patient gender.

Several reasons motivated our decision to focus on Hispanic adolescents: First, national surveys indicated that minorities are more likely to be affected. Nevertheless, it seems that risks are different depending on nation of origin. Compared to Mexicans, “other” Hispanic groups had higher risks of sleep debt (<5 hours per night). Second, Hispanics represent a sizable and rapidly growing subgroup of the U.S. population that has been understudied. Third, from the public health perspective, it is imperative to confirm if prior findings apply to this minority group of adolescents, as they are a rapid expanding population. Therefore, there is a need to understand which factors impact healthy sleep habits to integrate these unique issues (e.g., psychosocial) into preventive programs. Our *a priori* hypothesis was that Hispanic females would have a less favorable sleep profile, particularly if they were recent immigrants.

## 2. Methods

### 2.1. Study Design and Inclusion Criteria

ROBIM is a 5-year longitudinal study based in South Florida. This article focuses on the responses of the 500 Hispanic adolescents, which the American Academy of Pediatrics defines as minors 11–18 years old.

Youths were recruited through direct outreach in centers and health care facilities that provide recreational, social, and educational services for Hispanics. Adolescents were eligible if they did not have a history of a major neurological/psychiatric disorders (i.e., autism, severe developmental problems, mental retardation, schizophrenia), or clinical disease (i.e., cancer, renal, or heart disease) that prevented their participation in the study. Adolescents were ineligible if they received any neuro-pharmacological intervention or were taking bodybuilding substances (i.e. steroids, growth hormones).

After a complete description of the study to the adolescent and his/her legal guardian, written, informed consent was obtained from both parties. Only 25 adolescents were invited to participate, but were not enrolled because of schedule conflicts.

### 2.2. Assessments

ROBIM's baseline visit was conducted by trained interviewers, and consisted of a brief medical exam, structured survey questionnaires to obtain sociodemographic information, health behaviors (diet, exercise, sleep, drug use), and medical information (e.g., medical history, and medication use). As part of the protocol approved by the Florida International University and the University of Miami IRBs, parents completed brief questionnaires regarding sociodemographics, adolescent's health, including exposure to alcohol or drugs during pregnancy, and to provide a blood sample to test their BDNF levels.

### 2.3. Sleep

To assess sleep duration, teens were asked to indicate the time they go to sleep on regular school nights (Monday to Thursday night), and the time they get up in the morning on regular school days (Monday to Friday morning). Time in bed (TIB) was calculated by subtracting bedtime from rise time. Difficulties initiating sleep were rated on a three-point Likert scale, with response options of “not true”, “somewhat true” and “certainly true”. If a positive response was given (2 or 3 on the scale), the participants were asked about frequency in the past month (yes/no DSM-IV criteria).

According to national guidelines, teens <12 years old need 10–11 hours of sleep, while adolescents ages 12–18 need between 8:30 and 9:30 hours each night [9]. Based on this criterion, adolescents were classified as sleep deprived or not.

### 2.4. Independent Variables

Sociodemographic information was collected using both adolescent and parent self-report questionnaires. Participants were asked both their age and their date of birth. Since gathering information on physical, sexual, cognitive, and social development is complex, for pragmatic reasons, we used chronological age to define development boundaries. Children ≤12 years old were classified as preadolescents, with adolescents classified as 13–18 years old. Ethnicity was determined using participants' self-identification, country of origin, and the ethnicity of their parents and grandparents.

Adolescents were also asked about the place of birth (City and Country) and followed with a question about when they arrived to Florida if they indicated that they were born outside Florida. Based on their responses, the participants were classified as an international immigrant, US-born in Florida, and US-born in another state (internal migration).

Self-reported gender, ethnicity, number of years living in the US, and number of family members were obtained using standardized surveys. The surveys also enquire whether a male or a female was the head of the household and then about parent's income, employment, and education.

#### 2.4.1. Control Variables

We gathered information on variables known for affecting sleep, such as sociodemographics, neighborhood, and acculturation (measured by the Marin acculturation short scale) [10]. We also included indices of general health (medical history, medication intake, and body mass index), and health behaviors (e.g., caffeine consumption, drug use, and exercise via Stanford 7-day survey) [11].

#### 2.4.2. Statistical Analyses

Descriptive statistics were used to summarize the data. Group comparisons were assessed using the chi-square test for categorical variables, ANOVA for normally distributed variables, and the Wilcoxon rank sum test for nonparametrically distributed, continuous variables. Logistic regression modeling was used to evaluate the relationship between the main variables of interest (gender, migration, and development stage) and sleep outcome. Models included adjustments for other sociodemographic variables of the adolescents and the parent, country of origin, health behaviors, neighborhood stressors (environment), and acculturation.

## 3. Results

### 3.1. Sleep and Sociodemographic

The demographic characteristics of the adolescents currently enrolled in ROBIM are shown in Table 1. The age of the respondents ranged from 11 to 18 years, and the mean was 14.7 ± 2.0. The majority was either in middle or high school (35% and 50%, respectively), with the remaining participants being either in elementary school (14%) or in university (1%).

In this relatively large sample of healthy, typically developing adolescents, the average total sleep time during weekdays was 8.0 ± 1.6 hours (3–15 hours). Only 25% reported sleeping over 8.0 hours on school nights, and this rate increased to 35% each weekend. On workdays, the average bedtime was 10:55 PM compared to 1 AM during weekends.

In 15% of the adolescents, insomnia was the cause for suboptimal hours of sleep (6 vs. 8.5 hours/night), with all teens reporting difficulty initiating sleep (DIS) subtype.

We further sought to understand whether different patterns were the result of disparities in sociodemographic, language acculturation, and/or sleep environmental factors such as number of siblings (overcrowded environment) or neighborhood conditions. All analyses performed demonstrated that they were not significantly different.

### 3.2. Sleep Deprivation and Development Stage

The prevalence of sleep problems was age dependent, with 65% of the preadolescents reporting less than the recommended sleep time. An additional 5% reported sleeping more than 11 hours, suggesting a deteriorated quality of nightly sleep. When analyzing the older group, almost half had sleep deficits (45%, <7 hours nightly).

Participants seemed to try compensating for weekday sleep debt by oversleeping 1-2 hours on the weekend. Up to 34% of adolescents had a weekend bedtime delay of 2 hours or more, which can indicate a Delayed Sleep Phase disorder.

Since prior analyses suggested that overweight and obese children sleep less, we pursued such analyses. As depicted in Table 1, over half of the sample was overweight. Nevertheless, in normal-weight, overweight, and obese groups, comparisons of sleep values showed no significant differences either for weekdays or weekends. On the contrary, preadolescents who were obese slept more hours during weekdays (10.3 ± 1.9 vs. 8.5 ± 1.4 vs. 8.3 ± 1.3 hours, *p* = 0.03). Hours of sleep were very similar among all adolescents (7.8 ± 1.7 vs. 8.0 ± 2.0 vs. 7.7 ± 1.7 hours, *p* = 0.88).

### 3.3. Sleep Gender Differences

**Figure 1:**
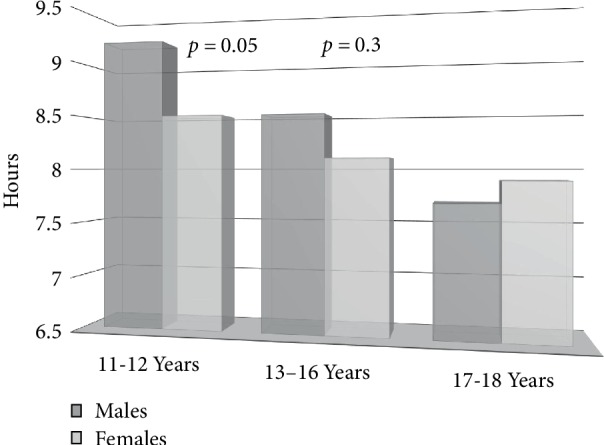
Sleep time by gender & age group.

The male to female ratio was nearly 1 : 1. Of convenience for these analyses, females at baseline had normal menstrual cycles, and consumption of coffee, alcohol, or drugs was minimal (10%). Gender differences in sleep deprivation and insomnia rates, according to DSM-IV criteria, were explored. At first glance, we did not observe significant gender differences in sleep measurements (8.7 ± 1.5 vs. 7.6 ± 1.7 hours, *p* = 0.2). Yet it needs to be acknowledged that females were sleeping less than 8 hours, which heightens the risk of chronically insufficient sleep. Since lack of differences may be a function of the pubertal status of the females sampled, which can be affected by the age ranges studied, we performed additional analyses. As depicted in Figure 1, males slept on average at least one more hour per day than their female counterparts, and when accounting for age, ANOVA became significant. Nevertheless, when teens reached the last year of high school, or the first year of college, they returned to sleeping a similar number of hours. Findings probably reflect the more intense pace of social life for older males.

Gender differences were evident, with 10% of males reporting insomnia versus 16% of females, with the risk of having insomnia nearly twice as high among females (OR = 1.8, 95% CI: 1–3.6, *p* = 0.05). No gender difference in circadian disorders was observed.

### 3.4. Sleep Deprivation, Family, and Environment

The study successfully recruited a sample of both high (34% < 80,000) and middle/low-income participants. Although sleep deprivation has been related to overcrowded environments and poverty, there were no statistically significant differences in the number of siblings between the groups. No relationship was observed between greater risk of insufficient sleep and being part of a family with low income and lower educational attainment in our study. Moreover, a tendency was observed for sleep deprived teens to be wealthy and not working at baseline.

With regard to the environment, the relationship between neighborhood stressors and weeknight and weekend sleep schedules was only marginally related (*p* = 0.08). Although total neighborhood scores were similar, prior data indicate that concerns for safety cause a state of chronic hyperarousal, so we pursued additional analyses. Indeed, we found that presence of gangs and hearing gunshots were associated with going to sleep late (12.20 ± 2 vs. 10.20 PM ± 2, *p* = 0.005).

### 3.5. Reported Causes by Gender

The sleep problems were rooted in several overlapping causes. The most common reason (40%) for sleep loss was electronic media use in the bedroom, (i.e., surfing the Internet, looking at TV/Netflix movies, or playing video games). Gender differences were evident in the proportion of males reportedly using a game console in the bedroom (11% vs. 1% females), and all males said they made at least one friend through online gaming, compared to none of the females. Socializing was second on the list (28% via cellular phone). Some identified (14%) striving for good grades or greater reliance on long work hours as a cause. A higher proportion of females than males (20.5% vs. 15%) reported sleeping less because of work. Others (10%) reported problems decreasing vigilance and arousal, due to anxiety and ruminative thinking about stressors.

### 3.6. Reported Causes by Gender and Migration Status

Most adolescents were native-born Americans, and 27% were born abroad. Of those born abroad, almost half were Cubans (43%), followed by Colombians (13%), Central Americans (Panama, Honduras, Guatemala, Nicaragua) (14%), Argentinians (9%), other South American (6% Uruguay, Peru, Ecuador, Venezuela), and Dominican Republic (6%).

Based on prior studies suggesting that immigrants sleep more hours than US born Hispanics, we contrasted their sleep hours during weekdays and weekends and did not find any significant differences. We also examined the impact of place of origin and found no significant differences. Yet, additional analyses provided a new perspective to these findings. For instance, Hispanic males born in the US reported going to sleep later during weekdays than females (12.0 ± 2 vs. 10.0 ± 1, *p* = 0.05), but differences disappeared during the weekends (11.5 ± 1 vs. 12.5 ± 1, *p* = 0.4).

On the contrary, female immigrants tended to go to sleep later during weekdays than their male counterparts (11.0 ± 2 vs. 9.0 ± 1 PM, *p* = 0.09), but woke up later during the weekends (11.0 ± 2 vs. 10 ± 1 AM, *p* = 0.03). As depicted in Table 2, when exploring the reasons driving sleep debt, more differences emerged. The males born in the US were the group most frequently citing playing video game as the primary reason for sleeping fewer hours than recommended. They were also more likely to report playing games online with friends, while none of the females reported this trend.

Two main reasons drove sleep debt in this group of females, having to work and insomnia. Notably, quite the opposite occurred among immigrants, males were more likely to report working as a cause. In contrast to females born in the US, only one female immigrant reported insomnia as a cause of sleep deficiencies.

### 3.7. Final Analyses

In order to clarify potential influences of social, cultural, acculturation, stress, and other behavioral risk factors on sleep, we performed a multivariate analysis. As depicted in Table 3, multivariable adjusted logistic regression analyses showed that females, US-born, and preadolescents had higher odds of being sleep deprived.

In Table 4 we show the results of the analyses for insomnia, in which the only significant predictor was the number of years since immigration to the US. Yet, acculturation was not a predictor. Age and gender were significant, but once immigration was included in the model, they were no longer significant.

## 4. Discussion

Although it is well established that adolescents in the United States have poor sleep habits, the vast majority of the studies are based on nonHispanic whites and to lesser extent African Americans. Limited research has been done in US Hispanics, and those available are mostly based on adults, which prevent making or extrapolating accurate conclusions [14–16]. Our analyses confirmed that these adolescents obtained less than the recommended hours of sleep for their age on school days. Our data discovered that our pre-adolescent Hispanics exhibited excessively high rates of sleep deprivation [1, 17]. Pre-adolescence seems to be a particularly risky developmental period for inadequate sleep patterns. Sleep hygiene practices were moderately poor among adolescents. Emphasizing good sleep hygiene practices and integrating sleep promotion programs into daily routines should be considered in order to improve the health of these adolescents.

### 4.1. Contribution to the Extant Literature

The obvious question is whether these excessive rates are due to factors amenable to change with target interventions. For example, is this due to increasing use of technology? While nearly all American adolescents have and use the computer, the internet, console games, and cellular phones, males and females use them at different rates as indicated by our analyses. Data are in line with prior surveys indicating that males playing video games do experience reduced subjective sleepiness, and experience slight sleep onset latency [12]. Based on these outcomes, additional studies are clearly justified.

Findings complement prior studies by examining immigration status, since family background can distinctively impact sleep behaviors. Indeed, female adolescents were more likely to report working as a cause for sleep deprivation. For immigrants, expectations of male gender roles may explain why more male immigrants reported working as a reason for sleeping fewer hours. Findings are in line with cultural ecological frameworks that recognize that behaviors need to be appreciated within a larger sociocultural context [10]. These results highlight the importance of not generalizing results to youth from other cultural backgrounds, even if they are from the same race. Notably, differences were driven by length of US residency, and not by the country of origin or the phenomenon of acculturation, as they do not modify the results of these analyses, whereas a prior analysis of sleep patterns in adults found that acculturation accounted for differences in sleep duration [14, 15].

The immigrant health advantage was also observed for insomnia [15]. Although we expect that stressors, work, and the familism typical of Hispanics may exert an effect on sleep, the lack of significance in the logistic models reflects that the underlying sources of the association between immigrant status and sleep are mediated by other variables and require further investigation.

To note in our final model, neither overweight nor obesity predicted the duration of sleep, although obese preadolescents seem to be sleeping more. These results suggest that the heterogeneity of published results regarding the relationship between sleep duration and obesity probably reflects sampling bias, or lack of consideration for the development stage [17]. On the other hand, our analyses confirmed the trend to sleep more during weekends, possibly as a compensatory mechanism to mitigate the adverse effects of a poor weekday-sleep pattern.

### 4.2. Limitations

Our findings need to be analyzed in the context of some study limitations. First, our study was limited to subjective sleep measures. We also recognize that the sample size although large (*n* = 500), may be insufficient to conduct statistical analyses by country of origin.

The strengths of our study are the use of a diverse sample of Hispanic adolescents, with a sizable proportion of females, and controlling for important demographic and clinical confounding factors.

In summary, while the widespread use of technology is a pervasive factor impacting adolescents' sleep, their impact differs by gender and immigration status. On the other hand, we are less clear about other factors that may affect sleep, such as job and neighborhood safety.

## Figures and Tables

**Table 1:** Demographic characteristics of the adolescent population (*n* = 500).

Demographic variable	Percent
*Gender*	
Male	47%
Female	53%

*Age*	
In years	14.7 ± 2

*Body mass index*	
14–25	30%
25–30	61%
>30	9%

*Current education*	
Elementary	14%
Middle school	35%
High school	50%
University	1%

*Income*	
Low/poverty	42%
Middle	26%
High class	32%

*Migration status*	
Immigrant	27%
Born in Florida	64%
Migrate from another US state	9%

Demographic characteristics of the total group expressed as percentages from all 500 participants from the ROBIM study.

**Table 2:** Reasons for sleep debt based on gender and migrant status.

		Socializing	TV/computer	Video games	Work	Insomnia	
US born	Males	33%	34%	14%	9%	10%	0.02
	Females	30%	28%	1%	19%	22%	

Immigrant	Males	20%	27%	3%	27%	24%	0.03
	Females	32%	42%	3%	21%	3%	

**Table 3:** Multivariate analysis of sleep deprivation by age, gender, and migrant status.

Coefficients^a^						
Model		Unstandardized coefficients		Standardized coefficients	*t*	Sig.
		*B*	Std. error	Beta		
1	(Constant)	10.557	1.016		10.391	.000
	Age in years	−.134	.060	−.181	−2.235	.027
	Gender	−.531	.235	−.153	−2.256	.025
	Where were you born?	.543	.275	.167	1.974	.050
	Years living in the US	−.035	.035	−.088	−1.004	.316

^a^Analyses concluded that being US born, a female, or under 12 years old all predicted sleep deprivation.

**Table 4:** Predictors of insomnia.

Coefficients^a^						
Model		Unstandardized coefficients		Standardized coefficients	*t*	Sig.
		*B*	Std. error	Beta		
1	(Constant)	1.901	.144		13.188	.000
	Age in years	.014	.008	.106	1.803	.072
	Migration yes/no	.055	.033	.104	1.642	.101
	Number of years living in the US	−.010	.005	−.148	−2.190	.029
	Household/income	−.012	.009	−.062	−1.255	.210
	Neighborhood	−.007	.007	−.050	−1.018	.309
	Body mass index	−.001	.003	−.023	−.442	.658
	Gender	−.035	.030	−.058	−1.160	.247

^a^Dependent variable: insomnia.

## Data Availability

The data used to support the findings of this study may be released upon application to the Florida International University IRB who can be contacted at: Maria Melendez-Vargas, mdemelen@fiu.edu, (305) 348–8311.

## Abbreviations

DSM-IV: Diagnostic statistical manual 4
CDC: Center for disease control
ROBIM: The role of brain derived neurotrophic factor in decision making participants
TIB: Time in bed
ANOVA: Analyses of variance
DSPD: Delayed sleep phase disorder.
